# Antimicrobial resistance pattern of avian pathogenic *Escherichia coli* with detection of extended-spectrum β-lactamase-producing isolates in broilers in east Algeria

**DOI:** 10.14202/vetworld.2023.449-454

**Published:** 2023-03-16

**Authors:** Chahrazed Aberkane, Ahmed Messaï, Chafik Redha Messaï, Tarek Boussaada

**Affiliations:** 1 Department of Agricultural Sciences, DEDSPAZA Laboratory, Mohamed-Khider University, Biskra, Algeria; 2 Department of Agricultural Sciences, PIARA Laboratory, Mohamed-Khider University, Biskra, Algeria; 3 Laboratory of Research Health and Animal Production, High National Veterinary School, Algiers, Algeria; 4 Department of Biology and Agronomy, University Mohamed El Bachir El Ibrahimi of Bordj Bou Arreridj, Algeria; 5 Scientific and Technical Research Centre for Arid Areas (CRSTRA) Biskra, Algeria

**Keywords:** antimicrobial resistance, avian pathogenic *Escherichia coli*, broilers, colibacillosis, Eastern Algeria, extended-spectrum β-lactamase

## Abstract

**Background and Aim:**

Avian pathogenic *Escherichia coli* (APEC) is the causative agent of colibacillosis, one of the most prevalent bacterial diseases responsible for significant economic losses in the poultry industry worldwide. This study aimed to assess the antimicrobial resistance (AMR) patterns of APEC isolates recovered from poultry in east Algeria and estimate the prevalence of extended-spectrum β-lactamase (ESBL)-producing isolates.

**Materials and Methods:**

In the slaughterhouse of Batna City (Algeria), livers indicating colibacillosis were sampled from 204 suspected carcasses with growth retardation and generalized congestion. *Escherichia coli* isolation and identification were performed on MacConkey agar using conventional methods and the API 20E system. Antimicrobial resistance susceptibility was tested by the disk diffusion method according to the Clinical Laboratory Standards Institute Guidelines. Extended-spectrum β-lactamase detection was carried out using the double-disk confirmation test.

**Results:**

One hundred sixty *E. coli* isolates were recovered (one isolate per sample). Avian pathogenic *Escherichia coli* isolates showed high levels of resistance to ampicillin and tetracycline (100%), nalidixic acid (95%), ofloxacin (93.75%), doxycycline (91.87), ciprofloxacin (87.50%), trimethoprim/sulfamethoxazole (62.50%), gentamycin (32.50%), chloramphenicol (27.50%), amoxicillin/clavulanic acid (16.25%), colistin (14.37%), and nitrofurantoin (10.62%). All strains were multidrug-resistant to at least three antibiotics, and more than half (52.52%) of the isolates were resistant to at least seven antibiotics. All isolates were susceptible to ceftriaxone, ceftazidime, and aztreonam. Two *E. coli* isolates were ESBL producers (1.25%).

**Conclusion:**

Avian pathogenic *Escherichia coli* resistance to most antimicrobial agents used in poultry may lead to antimicrobial therapy failure.

## Introduction

Avian colibacillosis is one of the most important bacterial diseases in the poultry industry and is caused by *Escherichia coli* strains belonging to a pathovar termed avian pathogenic *E. coli* (APEC), a Gram-negative bacterium that belongs to the family of Enterobacteriaceae [1, 2]. APEC strains are responsible for localized or systemic infections having multiple forms and symptoms, such as septicemia, chronic respiratory disease, swollen-head syndrome, enteritis, cellulitis, salpingitis, omphalitis, pericarditis, peritonitis, and synovitis [3, 4].

Colibacillosis is considered an economically devastating disease in Algeria and worldwide, leading to huge losses translated by high mortality, decreased performance, condemnation of carcasses at slaughter, and increased preventive and curative costs [4–6]. Accordingly, antibiotic therapy plays a key role in colibacillosis infection treatment. Various antimicrobial compounds are applied, such as β-lactams (penicillin), tetracyclines, sulfonamides, aminoglycosides, and fluoroquinolones. However, the irrational and irresponsible use of antibiotics has led to the emergence of antibiotic-resistant *E. coli* [7–9].

Antimicrobial resistance (AMR) represents a significant threat to animals and humans, as these resistance genes can be transmitted to humans directly through direct contact with animals or their manures or indirectly through the food chain [10, 11]. Several studies worldwide performed multidrug resistance (MDR) *E. coli*-based analysis in broiler chickens, focusing on countries with no strict regulations limiting antibiotic use. For instance, Algeria has little information on the epidemiology of AMR of *E. coli* [12].

Therefore, this study aimed to determine the sensitivity to antibiotics of *E. coli* recovered from local poultry farms in east Algeria and investigate the presence of extended-spectrum β-lactamase (ESBL)-producing isolates.

## Materials and Methods

### Ethical approval

This study was conducted following the recommendations of the Institutional Committee for the Protection of Animals of the National Administration of Higher Education and Scientific Research of Algeria (98-11, Act of August 22, 1998).

### Study period and location

The study was conducted from July to December 2021Broiler carcasses were selected from flocks reared in east Algeria (Batna, Biskra, Skikda, Tebessa, and Guelma). The samples were processed at the laboratory of Algerian Center for Quality Control and Packaging (Biskra).

### Sample collection

A total of 204 broiler carcasses were selected from 36 flocks. Flock sizes ranged between 3000 and 5000 broilers in each poultry house. Five chickens from each poultry house with growth retardation or generalized carcass congestion were randomly selected and presented for autopsy in the slaughterhouse of the eastern poultry group (Batna). At autopsy, livers with a lesion indicating colibacillosis were removed from these carcasses. The samples were taken aseptically in sterile containers and transported instantly to the laboratory for further analysis.

### Bacteriological analysis

*Escherichia coli* isolates were recovered from flamed livers after enrichment in brain-heart infusion broth (Bio-scan, Algeria) at 37°C for 18–24 h, subcultured on MacConkey agar (Liofilchem, Italy), and incubated aerobically at 37°C for 18–24 h. Gram-negative, oxidase-negative, and catalase-positive typical colonies were identified using the API 20E system (Bio-Mérieux, France).

### Antimicrobial sensitivity test

Strains were submitted to susceptibility testing against 16 antibiotics using the disk diffusion method on Mueller–Hinton agar (MHA; Liofilchem) according to the guidelines of the National Committee for the Clinical Laboratory Standards Institute [13]. The following disks of antibiotics were tested (Liofilchem; HiMedia, India; Bioanalyse, Turkey): amoxicillin/clavulanic acid (AMC; 20/10 μg), cefotaxime (CTX; 30 μg), ceftriaxone (CRO; 30 μg), ceftazidime (CAZ; 30 μg), aztreonam (AT; 30 μg), ampicillin (AMP; 10 μg), doxycycline (DO; 30 μg), tetracycline (TE; 30 μg), chloramphenicol (C; 30 μg), gentamycin (CN; 10 μg), nitrofurantoin (NIT; 30 μg), nalidixic acid (NA; 30 μg), ciprofloxacin (CIP; 5 μg), ofloxacin (OFX; 5 μg), colistin (COL; 10 μg), and trimethoprim/sulfamethoxazole (SXT; 25 μg). *Escherichia coli* 25922 was used as the reference strain. The diameters of inhibition zones were interpreted by referring to the reading table of Enterobacteriaceae as recommended by the Standardization of Susceptibility to the National Scale Human and Veterinary Medicine (2014) 7^th^ edition.

### Search for ESBL: Synergy test

Extended-spectrum β-lactamase screening was carried out under standard antibiogram conditions, as recommended by Jarlier *et al*. [14], by depositing an AMC disk (20/10 μg) to 30 mm center-to-center of the CTX disk (30 μg) on MHA. The plates were incubated for 18 h at 35°C. The test was considered positive by the appearance of synergy in the form of a champagne cork between the AMC and CTX disks.

### Extended-spectrum β-lactamase double-disk confirmation test

The strains that showed a decrease in diameter compared to third-generation cephalosporins without a clear synergy were subjected to a double-disk confirmation test using a CTX disk alone and combined with an AMC disk applied on MHA. The plates were incubated for 24 h at 35°C. Isolates were considered ESBL when an increase of ≥5 mm in the inhibition zone of the combination disks compared to that of the CTX disk alone was observed.

## Results

Among 204 broiler chickens with pathognomonic lesions suspected of avian colibacillosis, 160 *E. coli* strains were isolated at 78.43%. In the disk diffusion test, as shown in Table-1, the highest prevalence of resistance was found against AMP and TE (100%), followed by NA (95%), OFX (93.75%), DO (91.87%), and CIP (87.50%). Moderate resistance was noted for SXT and CN at 62.50% and 32.50%, respectively. Chloramphenicol, AMC, COL, and NIT had low resistance rates. All *E. coli* strains (100%) were sensitive to CRO, CAZ, and AT. Among 160 APEC isolates, only two strains were ESBL-positive.

**Table-1:** Antibiogram results for 160 APEC strains.

Antibiotic	*Escherichia coli* isolates (n = 160)			Frequency of resistance (%)

	S	I	R	
C, 20/10 µg	115	19	26	16.25
AMP,10 µg	0	0	160	100
DO, 30 µg	12	1	147	91.87
C, 30 µg	116	0	44	27.50
TE, 30 µg	0	0	160	100
CN, 10 µg	92	16	52	32.50
NIT, 300 µg	136	7	17	10.62
NA, 30 µg	8	0	152	95
COL, 10 µg	137	0	23	14.37
SXT, 25 µg	57	3	100	62.50
CIP, 5 µg	20	0	140	87.50
OFX, 5 µg	6	4	150	93.75
CTX30, 5 µg	100	0	60	37.50
CRO, 30 µg	0	0	0	0
CAZ, 30 µg	0	0	0	0
AT, 30 µg	0	0	0	0

R=Resistant, I=Intermediate, S=Susceptible, AMC=Amoxicillin-clavulanic acid, AMP=Ampicillin, DO=Doxycycline, C=Chloramphenicol, TE=Tetracycline, CN=Gentamycin, NIT=Nitrofurantoin, NA=Nalidixic acid, COL=Colistin, SXT=Trimethoprim/Sulfamethoxazole, CIP=Ciprofloxacin, OFX=Ofloxacin, CTX=Cefotaxime, CRO=Ceftriaxone, CAZ=Ceftazidime, AT=Aztreonam, APEC=Avian pathogenic *Escherichia coli*

In this study, all isolated *E. coli* showed MDR. They resisted at least three different antibiotics (Table-2), and 98.13%, 91.26%, and 80.64% of the isolates were resistant to four, five, and six antibiotic classes or molecules, respectively. More than half (52.52%) were resistant to at least seven antibiotics. A high MDR rate was recorded against nine antibiotics; only one isolate was resistant to 12 compounds tested. A total of 45 antibiotypes were obtained from APEC isolates. The most commonly found resistance patterns are listed in Table-3. The resistance profile (no. 3) was the most prevalent (16.87%), which was resistant to AMP, TE, NA, OFX, DO, CIP, and SXT.

**Table-2:** The prevalence of multidrug of 160 *E. coli* isolated from broiler chickens affected by colibacillosis.

Number of antimicrobial agent	Number of resistant *E. coli* strains	Rate of multidrug resistance (%)
1		
2		
3	1	0.62
4	2	1.25
5	11	6.87
6	17	1.62
7	45	28.12
8	62	38.75
9	17	10.62
10	3	1.87
11	1	0.62
12	1	0.62

*E. coli*=*Escherichia coli*

**Table-3:** Multidrug resistance patterns of the isolated APEC (n = 160).

No.	Antibiotic resistance profiles	Number of resistant strains (%)
1	AMP-TE-NA-OFX-DO	6 (3.75)
2	AMP-TE-NA-OFX-DO-CIP	7 (4.37)
3	AMP-TE-NA-OFX-DO-CIP-SXT	27 (16.87)
4	AMP-TE-NA-OFX-DO-CIP-CN	6 (3.75)
5	AMP-TE-NA-OFX-DO-CIP-SXT-CN	19 (11.87)
6	AMP-TE-NA-OFX-DO-CIP-SXT-C	10 (6.25)
7	AMP-TE-NA-OFX-DO-CIP-CN-C	5 (3.12)
8	AMP-TE-NA-OFX-DO-CIP-SXT-CN-C	9 (5.62)
9	AMP-TE-NA-OFX-DO-CIP-CN-C-COL-NIT	2 (1.25)
10	AMP-TE-NA-OFX-DO-CIP-SXT-C-AMC-COL-NIT	1 (0.62)
11	AMP-TE-NA-OFX-DO-CIP-SXT-CN-C-AMC-COL-NIT	1 (0.62)

AMP=Ampicillin, TE=Tetracycline, NA=Nalidixic acid, OFX=Ofloxacin, DO=Doxycycline, CIP=Ciprofloxacin, SXT=Trimethoprim/sulfamethoxazole, CN=Gentamycin, C=Chloramphenicol, AMC=Amoxicillin/clavulanic acid, COL=Colistin, NIT=Nitrofurantoin, APEC=Avian pathogenic *Escherichia coli*

## Discussion

Colibacillosis remains one of the most common, economic, and cost-effective bacterial diseases in the poultry industry, particularly among broilers globally and in east Algeria [5].

This study revealed that the highest resistance rate was recorded for AMP (100%). Similar results (89.70%) were obtained in the Algeria center [6] and in Pakistan [15], which examined the susceptibility of *E. coli* isolated from broiler flocks. However, another study in western Algeria [16] showed that *E. coli* strains had average resistance (62.50%).

In Table-1, APEC isolates were 100% resistant to TE. This finding was in accordance with Xu *et al*. [17], who reported that all APEC strains exhibited resistance to TE. In addition, Cheikh *et al*. [18] and Halfaoui *et al*. [19] demonstrated high resistance rates of 98.15% and 94.10%, respectively. This might be attributable to the inappropriate and accrued use of TE for treatment and prophylaxis in poultry farms. The resistance of *E. coli* to TE was produced by the acquisition and expression of *tetA* and *tetB* genes, as demonstrated by Van *et al*. [20]. The frequency of resistance was high (91.87%) for DO, probably because it shares a similar action mechanism to TE. Thus, acquired resistance to one automatically confers resistance to the other members of that family of antibiotics (cross-resistance) [21].

Moreover, high resistance rates to the quinolone group (95% for NA, 93.75% for OFX, and 87.50% for CIP) were shown in this study, consistent with Bakhshi *et al*. [22], who reported the highest resistance rate of APEC in Iran to NA (98.20%), followed by CIP (87.50%). Similarly, Merati *et al*. [23] in Tiaret Province showed that *E. coli* strains were entirely resistant to NA and OFX (100%). Regardless, these findings disagreed with a previous report in Uganda [24], which showed lower resistance to CIP and NA at 20.02% and 60.70%, respectively. The significant resistance to quinolones could be due to the excessive use of these compounds, as they have widespread availability in the Algerian market at a low cost. Fluoroquinolone resistance occurs through *qnrA*, *qnrB*, *gyrA*, and *gyrB* gene expression. Plasmid-mediated quinolone resistance promotes MDR propagation [2, 25, 26].

Resistance to SXT was lower than in previous results in Algeria and worldwide. Messai *et al*. [5] and Ibrahim *et al*. [3] found high resistance to SXT at 82% and 95.50%, respectively. Resistance to CN, C, and NIT was higher than in Benameur *et al*. [27], Halfaoui *et al*. [19], Boutaiba Benklaouz *et al*. [12], and Kazibwe *et al*. [24]. The administration of these antibiotics is forbidden in veterinary medicine in Algeria. Noticing a moderate resistance might be due to their illicit use, the persistence of resistance to these antimicrobial agents, and/or the phenomenon of co-selection.

Low resistance was noted to AMC (16.25%). Similar results were reported by Lounis *et al*. [6], who indicated a highly efficient level for AMC (7.70%). However, Radwan *et al*. [28] reported a high resistance rate to AMC (69.40%).

*Escherichia coli* tested in this study possessed a low resistance to COL (14.37%). These results agreed with the previous studies [12, 23] in western Algeria, describing that all APEC were susceptible to COL. These findings contradict the previous studies of Madadi *et al*. [29] and Azizpour and Ghazaei [30], who found significant resistance to COL. The low resistance to COL could be explained by the moderate use of this molecule in poultry, where it does not cross the intestinal barrier and is inactive orally on systemic colibacillosis. In contrast, the resistance of Gram-negative bacteria to COL is uncommon, even exceptional, and chromosome type. However, Garnacho-Montero *et al*. [31] reported that chromosomal mutation is rare.

Ceftriaxone, CAZ, and AT are considered the most efficient molecules. This result was anticipated as the cephalosporin group is not used in poultry flocks.

Positive ESBL strains showed resistance to TE, DO, AMP, and quinolones in this study. Boutaiba Benklaouz *et al*. [12] detected five *E. coli* strains belonging to ESBL producers. Another study by Meguenni *et al*. [32] in the center of Algeria showed 11 *E. coli* ESBL producers (CTX-M1 and CTX-M15) from tested isolates. Extended-spectrum β-lactamase-producing *E. coli* in chickens may result from abusing other antimicrobial agents. Moreover, ESBL occurrence was influenced by other factors, such as infection control measurements and antibiotic use [33]. Genes encoding for plasmid-mediated quinolone resistance are related to ESBL genes, as reported by Kim *et al*. [34]. Extended-spectrum β-lactamase-encoding genes in Enterobacteriaceae are often localized on plasmid vectors carrying genes responsible for the resistance to other families, such as those signaled by Perez and Bonomo [35] and Boutaiba Benklaouz *et al*. [12]. Maluta *et al*. [36] detected that APEC isolates are related to the human extra-intestinal pathogen *E. coli*. Chickens are a reservoir of ESBL that produce bacteria that can transmit to humans through direct contact, the food chain, or environmental contamination, as reported by Chabou *et al*. [37].

Multidrug resistance poses a serious threat as most isolates (100%) are resistant to at least three antibiotics (Table-2). Indeed, several antibiotics are administered often concomitantly for prophylaxis or in treatment. This indicates that the abuse and indiscriminate use of antibiotics is probably at the origin of the high incidence of antibiotic resistance and MDR of *E. coli* and the dissemination of resistance genes in the poultry industry in Algeria. Such practices, especially without prior antibiotic sensitivity testing of bacterial isolates, may lead to the development of a pool of antibiotic-resistant genes and the selection of increasing numbers of resistant *E. coli* clone genes.

This study considered the danger of APEC isolates expressing MDR profiles (nos. 3, 5, 6, 8, 2, 1, 4, 7, and 9). These strains could transfer their wide MDR pattern through the exchange of genetic material (conjugative plasmids or transposons). In this study, co-resistance to AMP-TE-NA-OFX-DO-CIP was noted in APEC isolates of all the most important MDR profiles, except the resistance profile (no. 1). Cantón *et al*. [38] reported that co-resistance is characterized by the transfer of numerous resistant genes into the same bacteria and by mutations in different genetic loci, affecting different antimicrobials. Harada *et al*. [39] demonstrated that MDR induces selection for resistance to antimicrobials that have not been used. Co-resistance to non-β-lactam antibiotics has been commonly reported in ESBL-positive *E. coli* isolates [40].

Antimicrobial resistance of *E. coli* infecting poultry has increased in recent decades, and antibiotic resistance has been changing in different parts of the world [14, 41]. Such resistant *E. coli* could spread to humans through chicken meat consumption and noncompliance with hygiene practices among farmworkers [42]. This is particularly significant in underdeveloped countries, where antibiotic-treated meat or other animal products should be avoided [43].

## Conclusion

Avian colibacillosis constitutes a real danger for mortality in the broiler sector, and ESBL production due to continuous use of antibiotics results in MDR and public health concerns. This study highlighted an alarming rate of *E. coli* strains resistant to most antimicrobial agents commonly used in poultry: AMP (100%), TE (100%), NA (95%), OFX (93.75%), DO (91.87%), CIP (87.50%), SXT (62.50%), and CN (32.50%). This study is phenotypical, and further molecular studies of these strains will make it possible to know them better and control this pathology. Thus, special programs are required to regulate antibiotic use in Algeria’s poultry sector and monitor the spread and transmission of APEC within the poultry industry. Furthermore, probiotics, organic acids, prebiotics, competitive exclusion, and vaccination should be investigated as viable alternatives to antibiotics to prevent treatment failures.

## Authors’ Contributions

CA, AM, CRM, and TB: Conceived and designed the study. CRM, AM and TB: Diagnosed the disease. CA, AM, CRM, and TB: Collected the samples and carried out the laboratory work. CA and CRM: Drafted the manuscript. CA, AM, CRM, and TB: Analyzed and interpreted the data. All authors have read, reviewed, and approved the final manuscript.
